# Effect of air pollution on age at menarche in polish females, born 1993–1998

**DOI:** 10.1038/s41598-022-08577-3

**Published:** 2022-03-21

**Authors:** Iwona Wronka, Katarzyna Kliś

**Affiliations:** 1 Laboratory of Anthropology, Institute of Zoology and Biomedical Research, Faculty of Biology, Jagiellonian University in Kraków, ul. Gronostajowa 9, 30-387 Kraków, Poland; 2 Department of Human Biology, University of Wrocław, Wrocław, Poland

**Keywords:** Developmental biology, Environmental sciences, Health care

## Abstract

The aim of the study was to analyse the association between the degree of air pollution (suspended particulate matter, sulphur dioxide, benzopyrene levels) in the location of residence during childhood and adolescence and the age at menarche. The research was carried out in the period from 2015 to 2018 in Poland. Anthropometric measurements were performed, and questionnaire data were collected from 1,257 women, aged 19–25 years. The average levels of subjects’ exposure to analysed air pollutants, i.e., particulate matter, sulphur dioxide, nitric oxide and benzene during childhood-adolescence was assessed from the data acquired by the Polish Chief Inspectorate for Environmental Protection. Negative relationships between age at menarche and suspended particulate matter as well as nitrogen levels were found. A similar trend was observed in an analysis of the relationship between age at menarche and the complex air pollution index. The tendency for age at menarche to decrease together with worsening air quality was also visible after adjusted for socioeconomic status. Girls exposed to high suspended particulate matter levels were characterised by higher risk of early age at menarche. High levels of air pollution are related to younger age at menarche and the risk of the menstruation onset below 11 years.

## Introduction

Ambient air quality is considered one of key determinants of human health^1,2^. Since biological development reflects an individual’s condition and is deeply affected both by chronic and frequent short-term illnesses, we may expect those differences due to ambient air quality will translate into variations in the rate of growth and maturation. In addition, air pollutants contain endocrine active substances which may impact human development and lowering the age at first menstruation in girls^3^. To date, however, those issues were analysed in only a small number of published studies^4–14^. This is still a topical and important research issue, as many children from different countries are growing up in a poor air quality environment.

Menarche is an important milestone in human biological development and is often considered as fertility signal. Girls experience menarche at different ages but usually it occurs between the ages of 10 and 16 years. Besides genetics, menarche is also influenced by socioeconomic and environmental factors^2^. Numerous studies analyse factors modifying the age at menarche, however, data on the impact of environmental pollution on the onset of menstruation is limited. To the best of our knowledge, such research has not yet been carried out in any European country.

It seems reasonable to investigate this problem, as many studies have shown a significant association between age at menarche and health status in adulthood. Menarche occurs between the ages of 10 and 16 years in contemporary girls from developed countries. Age at menarche below 12 years is usually classified as early, although some researchers set it at 11 y. or even at 10 years. The age of menarche above 14 years as late^15–18^.

Late menarche is associated with osteoporosis and increased fracture risk^19^. The early onset of menstruation increases the risk of obesity and numerous conditions such as breast and ovarian cancer, the metabolic syndrome, cardiovascular diseases, and hypertension^20–23^. Additionally, over the past 150 years a tendency for a decreasing age at menarche has been observed. Numerous studies indicate that the consequence of this phenomenon is the rise in the incidence of lifestyle diseases and the obesity epidemic^24,25^.

The aim of the study was to analyse the association between the degree of air pollution (suspended particulate matter, sulphur dioxide, nitric oxide, benzene levels) in the location of residence during childhood and adolescence and the age at menarche.

## Material and methods

The research was carried out in the period from 2015 to 2018 in Poland, among 1257 female university students aged 19–25 years (girls born in the years 1993–1998). The study protocol was approved by the Bioethics Committee of the Jagiellonian University in Kraków (opinion no. 122.612047.2016). Data were collected following the ethical principles as stated in the Declaration of Helsinki. Informed consent was obtained from all individual participants included in the study.

Age at menarche was assessed based on the date provided by the subjects. Students were asked to provide the date of their first menstruation with the accuracy within one month. The age at menarche was calculated from the date of menarche and the date of birth. In cases when, instead of an exact date, participants reported only month and year of menarche, the 15th day of an indicated month was used for calculation, and when the date of menarche was reported approximately in the range of 2–3 months, the midpoint of the reported period was used for calculation.

### Assessment of exposure to air pollution

Air quality was be determined based on the data made available by the Chief Inspectorate for Environmental Protection. The level of each analysed airborne pollutant, i.e.: particulate matter (PM_10_, PM_2.5_), sulphur dioxide (SO_2_), nitric oxide (NO) and benzene (C_6_H_6_) was considered as a categorical variable, which allowed to include in the assessment of exposure not only not only the average annual concentration levels of a given pollutant, but also the number of days per year with the daily limit exceeded. Based on data from 1995 to 2017 years from air pollution stations nearest participants’ residential locations we divided childhood and adolescence place of living into 3 categories. Class 1 (low air pollution level) comprised zones where annual pollutant values and the number of days per year with exceedances were below the allowable limit; Class 2 (medium air pollution level)—zones with annual values below the permissible limit, but with the number of days of exceeding the norm above the limit, and Class 3 (high air pollution level) included zones above the limit. Detailed division criteria are presented elsewhere and in the supplementary material S1^5^.

Based on all the level of each pollutant a complex air pollution index was established. Similarly, to other study^26^, to assess the joint effect of air pollutants, principal component analyses were used to group pollutants (PM_10_, PM_2.5,_ SO_2_, NO, and C_6_H_6_) to represent a source-related mixture. Since the first component was relatively high, scores for the single component were used as an indicator of air quality. Tertiles of the component were used to identify three air quality classes: good, moderate or unhealthy. The method is detailed described in our previously paper^5^.

### Assessment of socio-economic status

Socio-economic status was included in the analysis as covariates and it was determined on the basis of variables considered reliable indicators of living conditions and lifestyle in Poland and Europe: degree of urbanization of the place of residence, father’s and mother’s education, number of siblings, and financial situation. The following categories were created: for the place of residence in childhood and adolescence: village, city up to 100,000 inhabitants, city above 100,000 inhabitants; for mother’s and father’s education: vocational, secondary, higher; for number of siblings: none, one, two, three or more. Financial situation during childhood and adolescence was assessed based upon subject’s responses to a survey question: ‘Do you consider the economic situation in your home as: below average and bad, average, good, very good, changeable and/or hard to specify’. As there was only one response in the last category, it has been omitted.

Additionally, based on all the above data, a SES evaluation index was established. The principal components analysis (PCA) was used. PCA is a dimensionality-reduction method and obtained the first principal component explains the largest fraction of the variance within the original data. The loadings of analysed variables on the SES index were as follows: 0.44—place of living, 0.52—mother’s education, 0.51—father’s education, 0.42—number of children in family, and 0.41—financial situation. The eigenvalue for first component reached 2.07 and explained 51.6% of common variation in SES. Tertiles of the component were used to identify three SES groups (low, medium, and high).

Descriptive characteristics of the subjects are presented in Table 1.

**Table 1:** Descriptive characteristics of the subjects.

	Category	N	%
**Pollutant**			
PM_10_	Low	193	15.35
	Medium	501	39.86
	High	563	44.79
PM_2.5_	Low	264	21.00
	Medium	486	39.66
	High	507	40.33
Benzene	Low	610	48.53
	Medium	288	22.91
	High	359	28.56
SO_2_	Low	609	48.45
	Medium	186	14.80
	High	462	36.75
NO	Low	443	35.24
	Medium	593	47.18
	High	221	17.58
**Socioeconomic variables**			
Urbanisation	Village	468	37.23
	Town	415	33.02
	City	374	29.75
Mother’s education	Vocational	273	21.72
	Secondary	377	29.99
	Higher	607	48.29
Father’s education	Vocational	482	38.34
	Secondary	352	28.00
	Higher	423	33.66
Number of siblings	0	195	15.51
	1	589	46.86
	2	300	23.87
	3 and more	173	13.76
Material conditions	Modest	60	4.77
	Average	346	27.53
	Good	649	51.63
	Very good	202	16.07

### Statistical methods

Statistical analyses were performed with the use of statistical software Statistica 13.0 (by StatSoft Polska). This manuscript is a continuation of our research analysing the impact of air pollution on biological development therefore, we used the same statistical methods as previously^5^. The Shapiro–Wilk test was applied for stature distribution normality assessment, and the Levene’s test for assessing the equality of variance of the analysed data. Air pollutions were analysed as categorical variables. In statistical analysis the generalized linear model (GLM) was applied. Analyses were conducted in three steps. Model 1 included the level of each analysed air pollutant: particulate matter (PM_10_, PM_2.5_), sulphur dioxide (SO_2_), nitric oxide (NO), and benzene (C_6_H_6_). Model 2 was adjusted for the degree of urbanization of the place of residence. In case of subject who moved during childhood or adolescence, the degree of urban development was calculated as the average category of all residences of the subject, weighted by the length of each residential period. Model 3 included as covariates all socio-economic variables: degree of urbanization of the place of residence, father’s and mother’s education, number of siblings, and financial situation. All adjustment covariates were selected a priori^5^.

To assess the effect of multiple-pollutant exposure on the age at menarche two-way analysis of variance and logistic regression were applied. Significance in all statistical tests was set at the level of at least p < 0.05^5^.

## Results

### Effects of air pollution on the age at menarche

Mean age at first menstruation was 12.76 years with a SD of 1.35 (ranged from 9.14 to 16 years); the median for the group was 12.78. With regards to single pollutants, significant differences in mean age at menarche were reported depending on suspended particulate matter and nitrogen levels. Mean age at menarche dropped as the levels of the pollutants increased (Table 2). A similar trend was observed in an analysis of the relationship between age at menarche and the complex air pollution index (Table 3). Post-hoc test results (Tuckey’s HSD) demonstrated significant differences both between the group from areas of good and unhealthy air quality (p = 0.0000) and between the group from areas of moderate and groups from areas of good (p = 0.0083) and unhealthy air quality (p = 0.0345). The tendency for age at menarche to decrease together with worsening air quality was also visible after subjects were divided into groups of various SES (Table 3).

**Table 2:** Age at menarche (mean ± standard deviation) in Polish females born in the years 1993–1998 in relation to air pollutants levels in the place of residence during childhood and adolescence.

	Pollutants level			p-values		
	Class 1 Low	Class 2 Medium	Class 3 High	Model 1	Model 2	Model 3
PM _10_	13.32 ± 1.45	12.68 ± 1.34	12.54 ± 1.28	**0.0000**	**0.0000**	**0.0003**
PM_2.5_	13.18 ± 1.39	12.79 ± 1.45	12.55 ± 1.17	**0.0090**	**0.0097**	**0.0089**
benzene	12.61 ± 1.38	12.91 ± 1.34	12.73 ± 1.30	0.1469	0.1520	0.1422
SO_2_	12.82 ± 1.37	12.75 ± 1.38	12.71 ± 1.30	0.3800	0.4201	0.3946
NO	12.98 ± 1.37	12.71 ± 1.35	12.62 ± 1.33	**0.0031**	**0.0026**	**0.0034**

p-values based on GLM models; 1-unadjusted; 2-model adjusted for the level of urbanization of dwelling place; 3-model adjusted for socio-economic factors (dwelling place. parents’ education, number of siblings and financial conditions).

Significant values are in bold.

**Table 3:** Age at menarche Polish females born in the years 1993–1998 in relation to air quality and socioeconomic status (SES).

SES	Air quality	N	Mean	sd	Result of MANOVA	Signifacantly differed groups^a^
Low		414	12.83	1.31	Main effects	
Average		423	12.79	1.25	F = 0.43	
Good		420	12.87	1.34	p = 0.6521	
	1. Good	361	13.07	1.34	Main effects	1–2
	2. Moderate	450	12.85	1.29	F = 1.519	1–3
	3. Unhealthy	446	12.58	1.16	p = 0.0000	2–3
Low	1. Good	126	13.11	1.33	Interactions	1–2
	2. Moderate	150	12.75	1.21	F = 0.78	1–3
	3. Unhealthy	138	12.62	1.28	p = 0.5391	
Average	1. Good	128	12.94	1.29		
	2. Moderate	144	12.89	1.25		1–3
	3. Unhealthy	151	12.55	1.22		2–3
Good	1. Good	107	13.17	1.24		
	2. Moderate	156	12.91	1.27		1–3
	3. Unhealthy	157	12.55	1.29		2–3

^a^Based on post-hoc Tukey HSD test.

### Effects of air pollution on the risk of early menarche

A logistic regression model was applied in the assessment of the risk of early first menstruation (age below 11) depending on air pollution levels (Table 4). Girls exposed to high suspended particulate matter levels, both PM_10_ (p = 0.0182) and PM_2.5_ (p = 0.01043) were characterized by higher risk of early age at menarche. OR values were calculated as 3.18 (95% CI 2.29–4.69) and 3.25 (95% CI 2.34–4.80) respectively, which suggests that the likelihood of accelerated age at menarche in the group living in areas with high levels of particulate matter was more than three times higher than in the group from areas with low levels of particulate matter (OR = 1, Ref).

**Table 4:** The risk of the onset of menstruation below 11 years in Polish females born in the years 1993–1998 in relation to the air pollution.

Pollutants level	OR^a^	95% CI	p-values
**PM**_**10**_			
Low	1 (Ref.)		
Medium	1.92	1.61–2.07	
High	3.18	2.29–4.69	0.0182
**PM**_**2.5**_			
Low	1 (Ref.)		
Medium	2.01	1.73–2.16	
High	3.25	2.34–4.80	0.01043
**Benzene**			
Low	1 (Ref)		
Medium	0.98	0.61–1.28	
High	1.11	0.90–1.64	0.1568
**SO**_**2**_			
Low	1 (Ref.)		
Medium	1.03	0.78–1.38	
High	1.22	1.01–2.14	0.1725
**NO**			
Low	1 (Ref.)		
Medium	1.06	0.55–1.22	
High	1.47	0.65–1.35	0.0844

^a^OR adjusted for socio-economic factors (dwelling place. parents’ education, number of siblings and financial conditions).

## Discussion

According to our study, we may conclude that air pollution is a significant modifier of the timing of sexual maturation. Our results revealed that accelerated first menstruation occurs in girls who in early childhood and adolescence lived in areas of poor air quality and high suspended particulate matter levels. To the best of our knowledge, only one previous study analysed the effect of pollution on age at menarche, reporting similar results. In Korean girls heightened PM_10_ level was associated with earlier age at menarche^27^. The literature of the subject also includes studies on other maturation indicators. McGuin et al. analysed the effect of traffic pollutants and found that girls living within 150 m from a main road, or a highway reached pubarche a younger age. No relationship between traffic pollutants and thelarche was found^9^. Meanwhile, research by Huang et al. demonstrated that exposure to PM_10_ delayed thelarche^8^.

Several studies analysed the influence of lead, which may be contained in suspended particulate. It was found that, unlike particulate, increased lead level in blood is related to later age at menarche^13,14^.

The biological mechanism in which suspended particulate affects age at first menstruation is still unknown. What we do know, however, is that those pollutants may induce oxidative stress, causing inflammations in respiratory and cardiovascular system^28^. Moreover, they may contain endocrine active substances, which affect e.g., nervous, endocrine or reproductive systems^29,30^. Endocrine disrupters may trigger the secretion of kisspeptin and initiate the secretion of gonadotropin releasing hormone, thereby promoting hypothalamic maturation and consequently earlier puberty^31,32^. It was also proved that some compounds found in particle matter, especially polycyclic aromatic hydrocarbons which mimic oestrogens, may cause the disturbances in hormonal function of the female reproductive system^33–35^.

It should be noted that the composition of suspended particulate matter may vary depending on location and that other airborne substances may exacerbate the adverse effect of particulate on the body. For this reason, further research on the subject is recommended, preferably in groups living in various geographic environments. Poland is a country with poor air quality, where pollution is generated mostly by burning coal and solid fuels in stoves. Smog forms usually in winter in frosty weather associated with high pressure systems. It is characterized by high concentration of particulate matter, especially PM_10_ and PM_2.5,_ as well as benzopyrene, with no increase in sulphur dioxide concentrations.

Our study shows that high levels of air pollution are related to younger age at menarche and the risk of the menstruation onset below 11 y. This finding may be helpful when providing an explanation of the relationship between age at menarche and health conditions in adulthood, e.g., conditions of respiratory, cardiovascular, hormonal and reproductive system. They also may prove helpful in disease prevention. It seems that one of the key elements in this area is to draw attention to ensuring appropriate conditions for biological development in childhood and eliminating factors which may accelerate or decelerate maturation and as a consequence affect biological condition of an individual in her adulthood.

The main limitation of our study is potential misclassification of exposure. The assessment of exposure to airborne pollutants was based on data averaged over the period of more than a decade for a given village/town/city district. Obviously, such evaluation is far from precise and constitutes a limitation of the study. Children are not exposed to pollutants only in their place of residence, but also in their school, during their travel to school and all other activities, such as recreational activities, etc. However, it is not possible to precisely determine exposure to harmful substances in the air for large groups and long periods of exposure. For this reason, records of air pollution from stations monitoring the nearest residential addresses are often used in epidemiological studies^36–38^.

## Conclusions

The air pollutants PM_10_, PM_2.5_, and NO assessed separately, negatively affect the age at menarche, also after standardization to confounders, such as the level of urbanization of dwelling place or other socio-economic factors (parents’ education, number of siblings and financial conditions). Additionally, high levels of air pollution were related to the risk of the menstruation onset below 11 y.

The significant differences in the age at menarche age were found depending on the air quality index, which reflects the combined effect of all pollutants assessed. Girls growing up in an area with unhealthy air quality had the onset of menstruation at a younger age than girls from areas with good and moderate air quality.

Our findings confirmed that air pollution is a significant endocrine disrupting factor. Negative effects of elevated levels of air pollutants can be seen as early as adolescence. Given the correlation between early menarche and the risk of various diseases, it can be expected that air pollution in the place of residence during childhood may also have long term negative health effects.

## Supplementary Information

Supplementary Table 1.
