# Supplementary material for: Effect of air pollution on age at menarche in polish females, born 1993–1998
Source: Sci Rep. 2022 Mar 21;12:4820. doi: 10.1038/s41598-022-08577-3 (PMC8938500; doi:10.1038/s41598-022-08577-3)
Supplement: Supplementary file 1 — Supplementary Table 1. [file 41598_2022_8577_MOESM1_ESM.docx]

Supplementary Table - Criteria for air quality assessment

| pollutants |  | Primary criteria  Annual mean | Secondary criteria |
| --- | --- | --- | --- |
| PM_10_ | low | <30 µg/m^3^ | Daily limit (50 µg/m^3^) not to be exceeded more than 35 times per year |
|  | medium | 20-29.99 µg/m^3^  or | Daily limit (50 µg/m^3^) to be exceeded more than 35 times per year |
|  |  | 30-40 µg/m^3^ | - |
|  | high | >40 µg/m^3^ | - |
| PM_2.5_ | low | <20 µg/m^3^ | - |
|  | medium | 20-25 µg/m^3^ | - |
|  | high | >25 µg/m^3^ | - |
| Benzene | low | <2 µg/m^3^ | - |
|  | medium | 2-5 µg/m^3^ | - |
|  | high | >5µg/m^3^ | - |
| SO_2_ | low | <10 µg/m^3^ | Daily limit (125 µg/m^3^) not to be exceeded more than 3 times per year |
|  | medium | <10 µg/m3  or | Daily limit (125 µg/m3) to be exceeded more than 3 times per year |
|  |  | 10-20 µg/m^3^ | Daily limit (125 µg/m^3^) not to be exceeded more than 3 times per year |
|  | high | 10-20 µg/m^3^  or | Daily limit (125 µg/m^3^) to be exceeded more than 3 times per year |
|  |  | >20 µg/m^3^ | - |
| NO_2_ | low | <20 µg/m^3^ | - |
|  | medium | 20-40 µg/m^3^ | - |
|  | high | >40 µg/m^3^ | - |
